# Suspected silent pituitary somatotroph neuroendocrine tumor associated with acromegaly-like bone disorders: a case report

**DOI:** 10.1186/s12902-024-01657-7

**Published:** 2024-07-23

**Authors:** Tongxin Xiao, Xinxin Mao, Ou Wang, Yong Yao, Kan Deng, Huijuan Zhu, Lian Duan

**Affiliations:** 1 Key Laboratory of Endocrinology of National Health Commission, Department of Endocrinology, State Key Laboratory of Complex Severe and Rare Diseases Peking Union Medical College Hospital, Chinese Academy of Medical Sciences and Peking Union Medical College, Beijing, China; 2 Department of Pathology, Peking Union Medical College Hospital, Chinese Academy of Medical Sciences and Peking Union Medical College, Beijing, China; 3 Department of Neurosurgery, Peking Union Medical College Hospital, Chinese Academy of Medical Sciences and Peking Union Medical College, Beijing, China

**Keywords:** Pituitary neuroendocrinal tumor, Silent somatotroph, Acromegaly, Skull lesion

## Abstract

**Background:**

Growth hormone (GH) positive pituitary neuroendocrine tumors do not always cause acromegaly. Approximately one-third of GH-positive pituitary tumors are classified as non-functioning pituitary tumors in clinical practice. They typically have GH and serum insulin-like growth factor 1 (IGF-1) levels in the reference range and no acromegaly-like symptoms. However, normal hormone levels might not exclude the underlying hypersecretion of GH. This is a rare and paradoxical case of pituitary tumor causing acromegaly-associated symptoms despite normal GH and IGF-1 levels.

**Case presentation:**

We report a case of a 35-year-old woman with suspicious acromegaly-associated presentations, including facial changes, headache, oligomenorrhea, and new-onset diabetes mellitus and dyslipidemia. Imaging found a 19 × 12 × 8 mm pituitary tumor, but her serum IGF-1 was within the reference, and nadir GH was 0.7ng/ml after glucose load at diagnosis. A thickened skull base, increased uptake in cranial bones in bone scan, and elevated bone turnover markers indicated abnormal bone metabolism. We considered the pituitary tumor, possibly a rare subtype in subtle or clinically silent GH pituitary tumor, likely contributed to her discomforts. After the transsphenoidal surgery, the IGF-1 and nadir GH decreased immediately. A GH and prolactin-positive pituitary neuroendocrine tumor was confirmed in the histopathologic study. No tumor remnant was observed three months after the operation, and her discomforts, glucose, and bone metabolism were partially relieved.

**Conclusions:**

GH-positive pituitary neuroendocrine tumors with hormonal tests that do not meet the diagnostic criteria for acromegaly may also cause GH hypersecretion presentations. Patients with pituitary tumors and suspicious acromegaly symptoms may require more proactive treatment than non-functioning tumors of similar size and invasiveness.

**Supplementary Information:**

The online version contains supplementary material available at 10.1186/s12902-024-01657-7.

## Background

Acromegaly is mainly associated with growth hormone (GH) hypersecretion caused by GH/somatotroph pituitary neuroendocrine tumors (PitNETs) [1]. Symptoms like facial changes and other acromegaly-related metabolic abnormalities are clues for the suspicion of acromegaly, but they are not mandatory for diagnosis [2, 3]. Biochemical tests, including serum insulin-like growth factor-1 (IGF-1) and nadir GH during oral glucose tolerant test (OGTT), are critical to diagnosing acromegaly. Patients with normal IGF-1 levels are usually considered acromegaly unlikely [1]. The cut-off of nadir GH in the OGTT is traditionally 1.0ng/ml, while increasing clinicians consider 0.4ng/ml as a better diagnostic cut point when using assays capable of detecting lower GH levels [1, 2]. However, these diagnostic criteria still have limitations. For example, mild acromegaly could exhibit nadir GH < 0.4ng/ml [4], and the levels of IGF-1 could also be affected by physiological and non-physiological factors [1].

Up to 30% of patients with PitNETs synthesizing GH show normal IGF-1 and nadir GH levels [5–7]. Accordingly, silent GH PitNET is commonly used to describe clinical and biochemical non-functioning GH immunostaining-positive PIT-1 (pituitary specific transcription factor 1) lineage PitNETs. [7, 8] Silent GH PitNET is a rare entity (about 2–4% of resected PitNETs), and they might have a younger onset age and a higher risk of recurrence compared with common non-functioning PitNETs [7, 9, 10]. A continuous spectrum may describe the PitNETs with different clinical presentations, serum hormone levels, and pathologic characteristics: functioning (typical symptoms and elevated hormones), whispering or subtle (subtle symptoms with elevated hormones), clinically silent (no symptom with elevated hormones), and silent (no symptom with normal serum hormones) tumors [6, 10]. In addition to typical acromegaly, the classification and surveillance strategy of cases with mild hormone elevation or associated symptoms is still ambiguous. Clinically silent cases with elevated GH and IGF-1 were occasionally reported [3, 11, 12], but no reports of cases with acromegaly-related symptoms and normal hormone levels have been published to our knowledge.

Here, we report a paradoxical case with suspicious acromegaly symptoms, associated complications, and pituitary macroadenoma. The serum IGF-1 was normal, and nadir GH was 0.7ng/ml. A GH-positive PIT-1 lineage pituitary neuroendocrine tumor was confirmed after surgery, with IGF-1 and nadir GH decreasing significantly. We consider this case an untraditional subtype within the spectrum of GH PitNETs, as it does not align with the classic definitions of either acromegaly or silent GH-PitNETs. We aim to highlight that acromegaly-associated presentations might occur in clinically subtle or silent somatotroph PitNETs that cannot be diagnosed as acromegaly.

## Case presentation

**Fig. 1:**
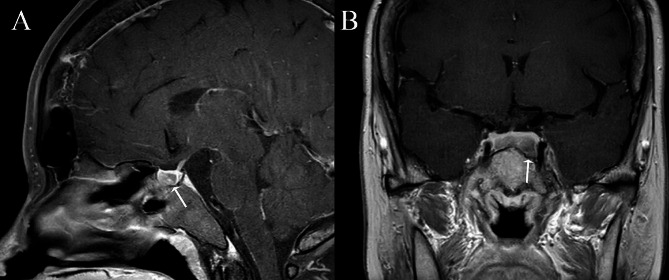
MRI image of the pituitary tumor. **A**, T1-weighted image (T1-WI) contrast-enhanced sagittal plane reveals a hypointense nodule, **B**, T1-WI contrast-enhanced coronal plane shows a macroadenoma across the right and left of the pituitary, without extension into the cavernous sinus or optical chiasm. Arrows indicated the location of the lesion

**Fig. 2:**
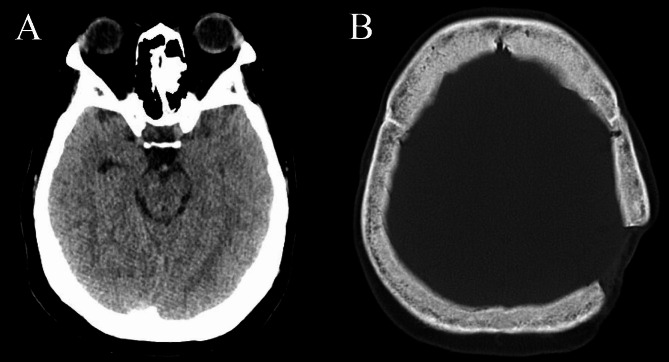
Head CT image showing a thickened skull base. **A**, Head CT image showed a thickened skull base; B, Head CT image showed a thickened skull base and the skull lesion caused by a previous traffic accident

A 35-year-old woman complained of oligomenorrhea and acromegaly-like facial changes of 13 years duration. She delivered a healthy baby following natural conception during this period, and her last menstruation was one year previously, after induced abortion for two consecutive pregnancy losses at eight weeks. She occasionally had headaches, excessive perspiration, and lower back discomfort, while she denied galactorrhea, taking contraceptives or any medications that may contain estrogen, and a familial history of pituitary disease. Her height and weight were 163 cm and 78 kg (with an increase of 23 kg since the onset of symptoms). She underwent surgery for a left occipital bone fracture because of a car accident at the age of 19, after which she gradually developed strabismus. The patient had late-stage pregnancy-induced hypertension, which resolved after delivery. She was diagnosed with diabetes mellitus one year ago but refused treatment. On physical examination, she showed widened nasal alae, thickened lip, and an enlarged tongue without enlargement of the hands and feet, suspected as acromegaly. Dynamic pituitary MRI found a 19 × 12 × 8 mm lesion in the pituitary gland without stalk deviation or extension into the cavernous sinus (Fig. 1). IGF-1 was measured twice with a 15-day interval, showing 212 and 133ng/ml (reference: 63–223ng/ml). The nadir GH after 75 g glucose load was 0.7ng/ml (Table 1). Other pituitary hormones stayed normal. Meanwhile, a thickened skull base was observed in CT (Fig. 2), and the whole-body bone scan indicated diffusely increased uptake in cranial bones. Bone turnover markers were elevated, with β-CTX and TP1NP measuring 2.06ng/ml and 135ng/ml, respectively. T-25OHD was 15.8ng/ml, and ALP was 213U/L. Her serum calcium, phosphate, and bone mineral density (Z-score at lumbar spine L1-L4: -0.7) were within the normal range (Table 1). These raised suspicion for metabolic bone disease. In addition, she had confirmed diabetes mellitus with Hb1Ac of 10.8% and hypertriglyceridemia with triglyceride rising to 3.96mmol/L. Hepatic steatosis was confirmed, with a mild impaired liver function (ALT ranged from 28 to 61U/L). A thorough body inspection did not find other suspected tumors. No pathogenic variants were identified by whole exome sequencing (WES), while two variants of uncertain significance were detected in the FOXA2 and LEPR genes (Supplementary Table 1).

**Table 1:** Clinical characteristics of the patient with suspected silent pituitary somatotroph tumor

Results	Reference	Pre-operation	3 days post-operation	3 months post-operation
IGF-1 (ng/mL)	63–223	212, 133	77	125
Random GH(ng/mL)	-	1.5	0.6	0.1
Nadir GH in OGTT (ng/mL)	-	0.7	0.4	-
F at 8 A.M. (µg/dl)	4.0-22.3	14.9	11.3	13.0
FT_4_ (ng/dl)	0.81–1.89	1.06	1.38	1.05
PRL (ng/ml)	< 30.0	11.5	6.6	11.0
Ca (mmol/L)	2.13–2.70	2.34	2.14	2.26
P (mmol/L)	0.81–1.45	1.33	0.95	1.08
ALP (U/L)	35–100	213	117	150
PTH (pg/ml)	15–65	37.5	39.6	84.4
T-25OHD (ng/ml)	> 30	15.8	-	7.3
β-CTX (ng/ml)	0.21–0.44	2.06	1.68	1.20
T-P1NP (ng/ml)	15.1–58.6	135	89.6	86.0

IGF-1: insulin-like growth factor-1; GH: growth hormone; OGTT: oral glucose tolerant test; F: serum cortisol; PRL: prolactin; Ca: serum calcium; P: serum inorganic phosphorus; ALP: alkaline phosphatase; PTH: parathyroid hormone; T-25OHD: total 25 hydroxyl vitamin D; β-CTX: carboxy-terminal cross-linked telopeptide of type 1 collagen; T-P1NP: total procollagen type 1 N-terminal propeptide

**Fig. 3:**
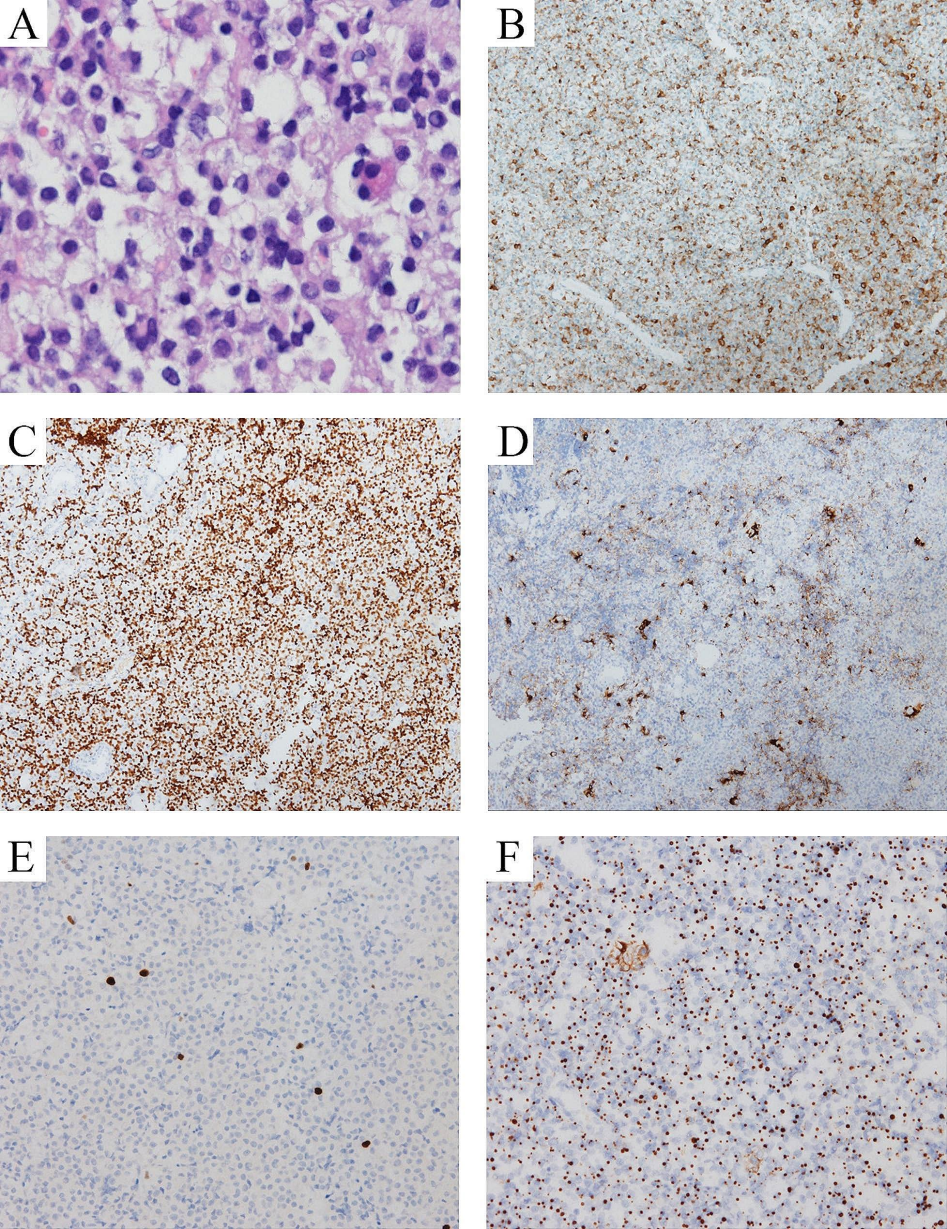
Pathologic and immunohistochemical image of the pituitary neuroendocrine tumor. **A**, H&E staining shows typical pituitary neuroendocrine tumor cells (×200). **B**, GH was moderately positive. **C**, PIT-1 was strongly positive. **D**, PRL was scattered and focal positive. **E**, ki-67 proliferative activity was approximately 1%. **F**, CAM5.2 was in a sparsely granulated pattern

In light of these suspicious symptoms, complications, and borderline nadir GH, we consider the possibility of a silent GH-secreting pituitary neuroendocrine tumor in this patient. Then, she received endoscopic transsphenoidal tumor resection surgery, with the soft pituitary tumor removed. In the histopathological examination of the resected specimen (Fig. 3), a PIT-1 lineage PitNET without other positive-staining transcription factors was confirmed. It was partially positive for GH while more weakly staining in PRL. Its Ki-67 proliferation index was 1%. CAM5.2 staining indicated a sparsely granulated pattern, and SSTR2 was positive. The staining of other pituitary hormones was negative. After the surgery, the IGF-1 dropped to 77ng/mL in 3 days and remained at 125ng/mL three months post-operation. Similarly, β-CTX and P1NP decreased significantly to 1.20ng/mL and 86.0ng/mL 3 months after surgery. (Table 1) She now takes one tablet of metformin sitagliptin twice daily and vitamin D3 1000U daily regularly. No new-onset discomfort was reported. 3 months after surgery, her fast blood glucose, Hb1Ac, and triglyceride were 7.9 mmol/L, 7.0%, and 3.41 mmol/L, respectively. No remnant of pituitary tumor was observed at the last visit.

## Discussion and conclusions

We report a paradoxical PitNET case showing several likely acromegaly-associated presentations but no elevation of IGF-1 or nadir GH after glucose load was confirmed. This patient experienced partial relief of discomforts after the pituitary surgery, and immunostaining showed a PIT-1 lineage tumor with moderately positive GH and a much weaker staining of PRL. We consider this case possibly an untraditional clinically silent GH pituitary PitNET.

In our case, both two random IGF-1 tests taken two weeks apart were in the normal range, with a borderline nadir GH. We observed a two-fold decrease in IGF-1 levels in tests taken two weeks apart, but this difference might simply be due to normal sampling and testing variations. Despite the limitations in biochemical diagnostic criteria for acromegaly and a possible weak impact of the metabolism of IGF-1 because of hepatic steatosis, it is the fact that these results could not distinguish the case from patients without GH PitNETs. However, this PitNET might still have a capacity for GH hypersecretion, even if it is relatively moderate. According to the theory of the continuous spectrum of PitNETs ranging from silent to functioning [10], the rising hormones may not cause obvious clinical symptoms [12, 13]. Meanwhile, it is widely accepted that clinical symptoms develop after the elevation of corresponding hormones. Still, our case showed a rare scenario in which a young woman had several acromegaly-like presentations with a normal IGF-1 and 0.7ng/ml nadir GH. Although her amenorrhea was likely due to uterine lesions, other symptoms like facial changes and comorbidities (including diabetes mellitus, dyslipidemia, and metabolic bone disease) were all possibly acromegaly-associated [1]. For example, acromegaly is associated with abnormal skeletal metabolism, leading to elevation of bone turnover markers, lower bone quality, and increased risks of fractures [14, 15]. In our case, decreasing bone turnover markers after pituitary tumor resection supported that the GH-positive PitNETs had at least a partial influence on her abnormal bone metabolism. Likewise, metabolic complications in this case, including diabetes, dyslipidemia, and hepatic steatosis, are also diseases recommended for screening in acromegaly [1]. The concurrence of these diseases is less likely to be a coincidence without an underlying hypersecretion of GH.

Meanwhile, differential diagnoses of a thickened skull base, increased radioactive uptake in a bone scan, and elevated bone turnover markers should also be considered. These differential diagnoses mainly included other metabolic bone diseases, like Paget disease of bone, osteopetrosis, fibrous dysplasia of bone, and pachydermoperiostosis [16]. However, this patient did not meet the clinical diagnosis criteria for most of these diseases. She had no enlargement of hands or hypertrophic skin changes, while WES did not identify any relevant gene mutation. Considering the rapid decline of IGF-1 and nadir GH after the surgery, together with her early-onset diabetes mellitus and hypertriglyceridemia without relevant familial history, from a monistic perspective, we suppose that it is highly likely that her GH-positive PitNET, which may cause a relatively moderate but long-lasting GH secretion, contributed most to the abnormal condition. Still, given the atypical and rare combination of active clinical symptoms and silent test results in this case, we conservatively considered that other comorbidities would not be completely ruled out at this stage. A long-term follow-up to observe whether her presentations and bone turnover markers could be relieved well without recurrence of the PitNET could assist in the final confirmation of the role of GH-PitNETs in this case.

As for pathologic classification, because of the predominant GH staining, a weaker PRL staining, and no TSH staining, this PIT-1 lineage PitNET was classified as a mammosomatotroph tumor. GH and PIT-1 positive PitNETs span a wide range of heterogeneous tumors with different clinical characteristics, like aggressiveness and secretion activity [8, 17]. Silent or subtle GH PitNETs share the pathologic classification with typical acromegaly, but their distribution of specific pathological subtypes differs. More tumors are likely expressing multiple pituitary hormones or even multiple transcription factors in silent PitNETs [7, 10]. For example, over half of silent GH PitNETs could have co-positive staining of PRL, as shown in our case, which is much more than those causing acromegaly [9]. Although the tumor, in this case, did not find invasion on imaging and its Ki-67 proliferation index was 1%, a close follow-up is recommended because it also exhibited several high-risk features.

For PitNETs that can express hormones like GH or ACTH without inducing noticeable hormone level elevations and symptoms, the underlying mechanisms remained unclear. An intriguing hypothesis is that some of these PitNETs might display a more primitive stage of differentiation [5]. Therefore, they may tend to retain the ability to express more pituitary hormones or even transcription factors, while hormone synthesis or secretion functions are less developed. This may also explain why PitNETs with higher aggressiveness are more common in silent GH or ACTH tumors [10, 18]. Another common assumption is that a short disease duration causes a lack of clinical change, especially when the secretion capacity of somatotroph PitNETs is moderate. However, a short disease duration could not explain our case since her clinical presentations seemed more apparent than the rechecked hormone levels at diagnosis.

Additionally, we propose another potential explanation for biochemically silent cases with GH hypersecretion symptoms: GH-positive PitNETs may also secret GH cyclically, similar to cyclic Cushing disease. There are reports of ‘silent’ corticotroph PitNETs showing associated manifestations without remarkable biochemical tests [19], but no similar case has yet been reported in somatotroph PitNETs. It is possible that our patient was tested at her trough in GH concentration, but the long-lasting effect of cyclic hypersecretion of GH and IGF-1 may cause associated symptoms. The mechanisms of cyclic Cushing disease are also undetermined. Hypotheses include hypothalamic dysfunction, the infarction or bleeding of pituitary tumors, and uncommon sensitivity to positive and negative feedback in specific patients [20, 21]. These assumptions may also be applied to GH-positive tumors. Since the half-life of IGF-1 is longer than serum cortisol in Cushing’s disease, a distinct fluctuation caused by ‘cyclic acromegaly’ would be more challenging to detect if such tumors indeed exist.

In summary, a normal IGF-1 and nadir GH at diagnosis cannot exclude the possibility of underlying GH hypersecretion from pituitary tumors in patients with suspicious acromegaly presentations. Tumors resection might improve acromegaly-like symptoms, and silent GH PitNETs have a higher risk of invasiveness and recurrence. Therefore, a more proactive surgical treatment should be considered in suspicious GH PitNETs than non-functioning tumors of similar size.

### Electronic supplementary material

Below is the link to the electronic supplementary material.

Supplementary Material 1

## Data Availability

No datasets were generated or analysed during the current study.

## Abbreviations

GH: Growth hormone
IGF-1: Insulin-like growth factor 1
PitNETs: Pituitary neuroendocrine tumors
OGTT: Oral glucose tolerant test
PIT-1: Pituitary specific transcription factor 1
PRL: Prolactin
β-CTX: Carboxy-terminal cross-linked telopeptide of type 1 collagen
T-P1NP: Total procollagen type 1 N-terminal propeptide
