# Supplementary material for: Suspected silent pituitary somatotroph neuroendocrine tumor associated with acromegaly-like bone disorders: a case report
Source: BMC Endocr Disord. 2024 Jul 23;24:121. doi: 10.1186/s12902-024-01657-7 (PMC11265331; doi:10.1186/s12902-024-01657-7)
Supplement: Supplementary file 1 — Supplementary Material 1 [file 12902_2024_1657_MOESM1_ESM.docx]

| Gene | Mutation Location | Gene  Subregion | Variant description | Zygosity | Variant Category | Associated Disease |
| --- | --- | --- | --- | --- | --- | --- |
| FOXA2 | Chr20:22582241-22582241 | exon2 | c.1001C>T:  p.A334V | Heterozygous | VUS | Pituitary hormone deficiency |
| LEPR | chr1:65592685-65592685 | exon6 | c.523G>A:  p.V175I | Heterozygous | VUS | Morbid obesity due to leptin  receptor deficiency (AR) |

**Supplementary Table 1**. Variants with Insufficient Evidence of Pathogenicity

VUS, Variant of Uncertain Significance. AR: Autosomal Recessive.

The reference database version is: Human Genome 38 (hg38/GRCh38).
